# Engineering Human Cells Expressing CRISPR/Cas9-Synergistic Activation Mediators for Recombinant Protein Production

**DOI:** 10.3390/ijms24108468

**Published:** 2023-05-09

**Authors:** Colby J. Feser, James M. Williams, Daniel T. Lammers, Jason R. Bingham, Matthew J. Eckert, Jakub Tolar, Mark J. Osborn

**Affiliations:** 1 Department of Pediatrics, Division of Blood and Marrow Transplantation, MMC 366 Mayo, 8366A, 420 Delaware Street SE, Minneapolis, MN 55455, USA; feser004@umn.edu (C.J.F.); tolar003@umn.edu (J.T.); 2 Department of General Surgery, Madigan Army Medical Center, 9040 Jackson Ave, Tacoma, WA 98431, USA; jmwill10@gmail.com (J.M.W.); dtlammer@gmail.com (D.T.L.); jrpbingham@gmail.com (J.R.B.); matteckert1@gmail.com (M.J.E.); 3 Department of Surgery, University of North Carolina, 160 Dental Circle, Chapel Hill, NC 27599, USA

**Keywords:** recombinant protein, cellular engineering, CRISPR/Cas9, multiplexing, clotting factors, fibrinogen

## Abstract

Recombinant engineering for protein production commonly employs plasmid-based gene templates for introduction and expression of genes in a candidate cell system in vitro. Challenges to this approach include identifying cell types that can facilitate proper post-translational modifications and difficulty expressing large multimeric proteins. We hypothesized that integration of the CRISPR/Cas9-synergistic activator mediator (SAM) system into the human genome would be a powerful tool capable of robust gene expression and protein production. SAMs are comprised of a “dead” Cas9 (dCas9) linked to transcriptional activators viral particle 64 (VP64), nuclear factor-kappa-B p65 subunit (p65), and heat shock factor 1 (HSF1) and are programmable to single or multiple gene targets. We integrated the components of the SAM system into human HEK293, HKB11, SK-HEP1, and HEP-g2 cells using *coagulation factor X (FX)* and *fibrinogen (FBN)* as proof of concept. We observed upregulation of mRNA in each cell type with concomitant protein expression. Our findings demonstrate the capability of human cells stably expressing SAM for user-defined singleplex and multiplex gene targeting and highlight their broad potential utility for recombinant engineering as well as transcriptional modulation across networks for basic, translational, and clinical modeling and applications.

## 1. Introduction

Peptides are employed for a multitude of therapeutic approaches and can be obtained from the native organ or tissue in which they are produced as well as through recombinant methods where nucleic acids encoding a candidate gene(s) are delivered to and expressed in cells in vitro. As an example, coagulation proteins such as factor X (FX) and fibrinogen (FBN), which are commonly employed for restoring hemostasis, are produced in the liver and secreted into the plasma [1,2,3,4,5]. In order to obtain sufficient quantities for therapeutic application that often requires multiple injections, plasma is pooled from multiple donors [6]. This requires a dedicated population of human donors necessitating stringent pathogen screening to ensure product safety, making plasma-derived acquisition methods costly and inefficient [7]. Alternatively, the ability to generate peptides through recombinant techniques in vitro represents an engineering approach for the production of these and other candidate peptides.

In vitro cellular candidates for recombinant production include prokaryotic and eukaryotic systems such as bacteria, yeast, and mammalian cells. Employing a cell type requires careful consideration of cell-specific capabilities and peptide functional properties. For example, tolerance of high-density culture, efficient gene transfer, and high-yield protein production are desirable host cell characteristics. A further guiding principle is the post-translational modifications (PTMs) that are present and required for the function of many proteins and whether a candidate cell possesses the requisite machinery to mediate PTMs.

Bacteria and yeast have many desirable cellular characteristics for recombinant protein production in that they can be grown to high density resulting in putatively high protein yields. However, potentially offsetting limitations include costly pathogen screening requirements, the formation of inclusion bodies that can limit protein yield, and a restricted PTM profile compared with eukaryotic cells [8].

Chinese hamster ovary (CHO) cells are a mammalian cell type that has been extensively used for recombinant protein production including the expression of human FX and FBN [9,10]. However, the glycosylation patterns carried out by CHO include the addition of N-glycolylneuraminic acid (Neu5Gc) [11]. This is a critical consideration given that most humans express anti-Neu5Gc antibodies and agents such as FX or FBN, which can contain these modifications, are often given in multiple doses potentially contributing to Neu5Gc-related immune sensitization and related side effects [11,12]. 

Most proteins possess PTMs, and these are often unique to a given organism complicating trans-species protein engineering approaches. Human embryonic kidney (HEK293), HKB11, SK-HEP1, and HEP-g2 cells have all been employed for recombinant protein production including for coagulation factors [13,14,15,16]. For example, Popovic et al. showed that HEK293 cells, known for their ease of culture and efficient gene transfer, can be employed for FBN production [13]. HKB11 cells, a HEK293 and B cell fusion, have demonstrated an increased capacity for protein expression and higher yields than HEK293 cells when utilized for coagulation factor production [14]. Similarly, HKB11 cells along with hepatocyte SK-HEP1 and HEP-g2 cell lines were used by Corrêa de Freitas and colleagues for coagulation factor production [15]. We reasoned that these cell types would benefit from a cross-platform comparison to define the optimal cell type and approach for recombinant peptide production using FX and FBN as proof of principle candidates.

These aforementioned studies advanced the field and their respective strategies employed plasmid-borne DNA expression elements. Plasmids are small circular DNA species that are produced in bacteria and delivered to cells with a concomitant increase in gene and protein expression. Plasmids can be expressed as either transient, non-integrating extrachromosomal episomes or as linear DNA for integration into the genome for stable expression. Transient approaches result in high copy number delivery; however, plasmid episomes are progressively lost as the cell divides. Alternatively, plasmid integration leading to stable expression occurs at a low frequency often requiring clonal isolation, expansion, and characterization [17]. The constituents of a plasmid for gene expression include regulatory elements (e.g., promoter and polyadenylation signal) and the candidate gene cDNA(s). For instance, FX is a monomeric peptide produced from a single gene while the fibrinogen peptide is trimeric with each subunit being encoded by a distinct gene. As such, the cargo needed to be delivered for multimeric peptide production strategies encompasses multiple cDNAs that can be delivered on separate plasmids or constructed into a single plasmid. The need to produce multiple plasmids or single, large plasmids prompted us to pursue a strategy whereby monomeric or multimeric proteins could be produced in human cells while minimizing the amount of plasmid required to be produced and delivered. 

We hypothesized that the powerful gene activation components from the CRISPR/Cas9-synergistic activator mediator (SAM) system [18] could be embedded into the genome of cells as an operating platform for candidate gene and protein upregulation. To accomplish this, we generated lentiviral particles containing a catalytically inactive Cas9 that is linked to transcriptional potentiators including Herpes simplex viral (HSV) particle 64 (dCas9-VP64), nuclear factor-kappa-B p65 subunit (p65), and heat shock factor 1 (HSF1) [18]. SAMs mediate endogenous candidate gene overexpression in the presence of a small guide RNA (gRNA) targeted to a region proximal to a start codon. We leveraged this approach and system for human and mouse cell engineering and showed single and multiplex gene expression of coagulation factor X and fibrinogen, respectively. SAMs are also broadly applicable to the fields of gene and cellular engineering for the modulation of genes, transcriptional network modeling, and basic and translational biology.

## 2. Results

### 2.1. Transgenic Human Cell Line Engineering for Stable Expression of the CRISPR/Cas9-SAM System and Application for Singleplex Gene and Protein Overexpression

**Figure 1:**
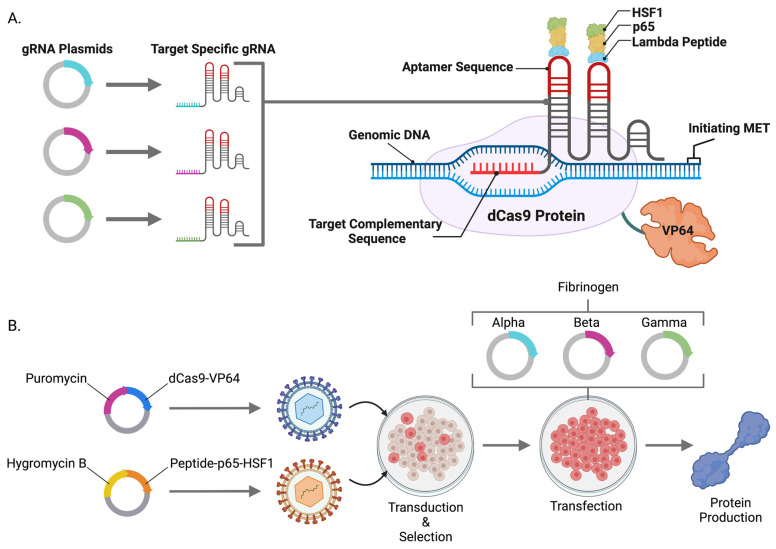
CRISPR/Cas9-Synergistic Activation Mediator (SAM) System Components. (**A**) Illustration of CRISPR/Cas9-SAM system component interactions and adaptability to user-defined genomic target sequences via minimal plasmids. (**B**) Cellular engineering strategy to integrate the central CRISPR/Cas9-SAM components into the genome of candidate cells.

The SAM system is comprised of “dead” Cas9 (dCas9) that forms a complex with a small gRNA transcript (gRNA) enabling programmable targeting of the complex to a user-defined locus (Figure 1A) [18]. dCas9 does not mediate DNA cleavage; instead, it has been fused to HSV VP64, a potent transcriptional activator [19]. To further potentiate gene expression, SAM gRNAs are uniquely engineered to include stem loops containing aptamer sequences that can bind a cognate peptide such as λ fused to p65 and HSF1 [18,20]. These domains can be programmed to a transcriptional start site for potent gene transcription [18].

In pursuit of our goal of engineering cell lines capable of supranormal gene expression, we constructed two lentiviral vectors with one carrying dCas9-VP64 and a second carrying the Lambda aptamer peptide/p65/HSF1 fusion (λ-p65-HSF1), each with a unique mammalian antibiotic resistance gene (Figure 1A). Vesicular stomatitis virus glycoprotein G (VSV-G)-enveloped lentiviral particles were produced and introduced into HEK293, HKB11, SK-HEP1, and HEP-g2 cells followed by drug selection to obtain transgenic cell populations (Figure 1B).

**Figure 2:**
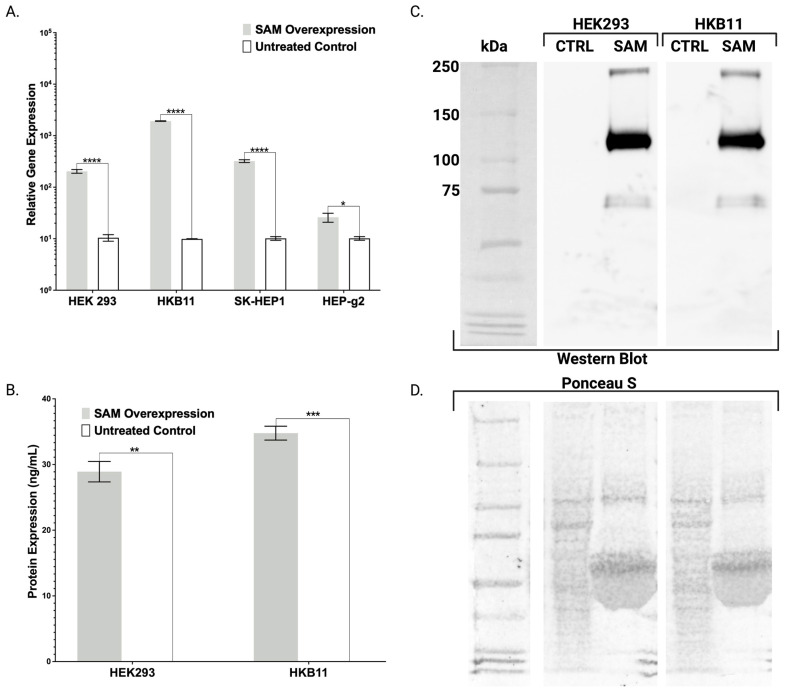
Engineered Transgenic Cells for Singleplex Overexpression of Coagulation Factor X. (**A**) mRNA-based gene expression analysis by qRT-PCR illustrating human *coagulation factor X* transcript expression in transgenic human cell lines (HEK293, HKB11, SK-HEP1, and HEP-g2) stably expressing SAM system components dCas9-VP64 and λ-p65-HSF1. Cells were either treated with two plasmids carrying the gRNAs to target human *coagulation factor X* or left untreated. (n = 3 independent experiments). (**B**) ELISA-based protein quantification of subsequent coagulation factor X overexpression in the conditioned media of treated or untreated transgenic HEK293 and HKB11 cells. (n = 2 independent experiments). (**C**) Western blotting and (**D**) Ponceau S staining of conditioned media comparing treated or untreated transgenic HEK293 and HKB11 cells. Error bars display standard error of mean. *p* values were calculated using Student’s *t*-test to compare treated and untreated cells (* *p* ≤ 0.05, ** *p* ≤ 0.01, *** *p* ≤ 0.001, and **** *p* ≤ 0.0001).

Stable cell lines were rapidly obtained (approximately two weeks) and to functionally assess their ability to mediate gene expression we transfected them with gRNAs targeted to bind proximal to the initiating methionine of the *FX* gene. We observed significant increases in *FX* mRNA expression with an average 3-fold increase in HEP-g2 cells, a 19-fold increase in HEK293 cells, a 31-fold increase in SK-HEP1 cells, and a 189-fold increase in HKB11 cells, compared with the corresponding untreated controls by qRT-PCR (Figure 2A). These data show the ability of transgenic human cell lines stably expressing the central SAM components of dCas9-VP64 and λ-p65-HSF1 to drive user-defined single gene expression when targeting gRNAs are delivered as plasmids. 

We then confirmed qRT-PCR data with protein analysis by ELISA and Western blotting. FX is a secreted peptide and media were collected from SAM HEK293 and HKB11 cells and in this cellular supernatant we observed yields of 29 +/− 2 ng/mL in HEK293 cells and 35 +/− 1 ng/mL in HKB11 by ELISA while control media had no detectable FX (Figure 2B). Subsequent Western blot analysis revealed protein bands from treated transgenic HEK293 and HKB11 cells consistent with the known multiple pre- and pro- isoforms containing numerous PTMs observed in FX (Figure 2C) [3]. No bands in the corresponding untreated controls were observed (Figure 2C). Importantly, the protein loading gel showed proteins for each sample (Figure 2D). These protein data demonstrate that the observed gene overexpression from transgenic CRISPR/Cas9-SAM human cells targeting *FX* translates into corresponding protein expression.

### 2.2. Engineered Transgenic Human Cells for Multiplexed Fibrinogen Gene and Protein Overexpression

**Figure 3:**
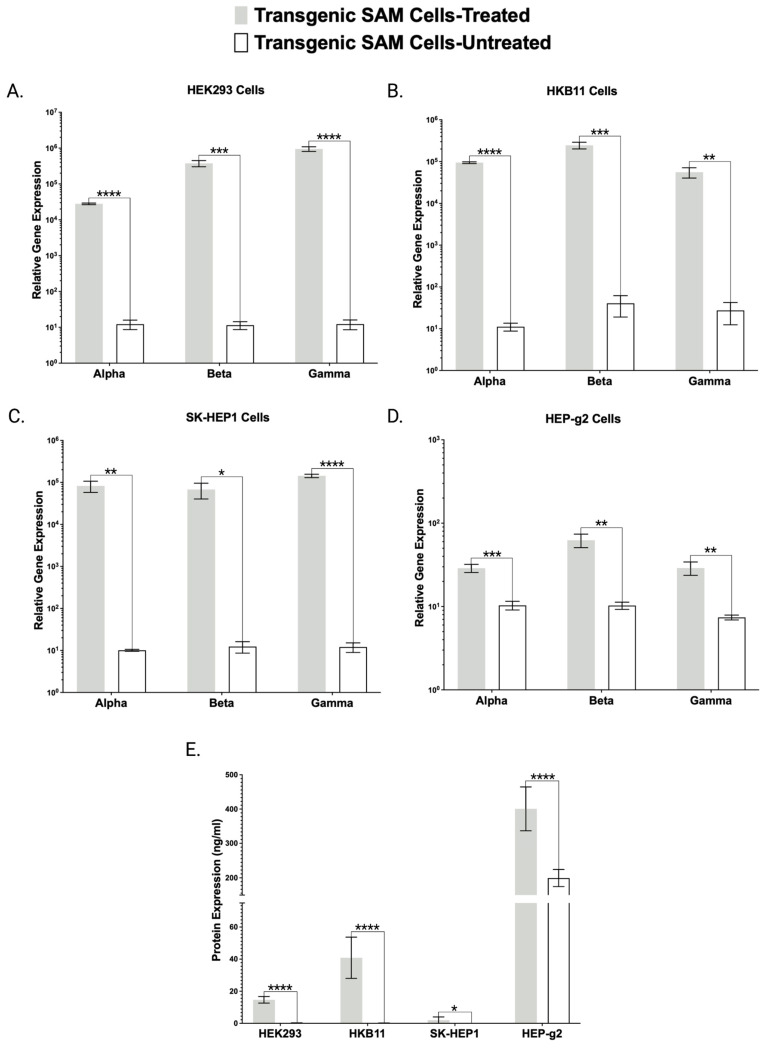
Transgenic Human Cell Lines Demonstrate Fibrinogen Gene and Protein Overexpression. mRNA-based gene expression analysis of *FBA*, *FBB*, and *FBG* by qRT-PCR from (**A**) HEK 293, (**B**) HKB11, (**C**) SK-HEP1, and (**D**) HEP-g2 cells. Transgenic cells stably expressing SAM components dCas9-VP64 and λ-p65-HSF1 were either treated or untreated with plasmids carrying the gRNAs to target *FBA*, *FBB*, and *FBG*. (**E**) ELISA-based protein quantification of subsequent FBN overexpression. (n = 3 independent experiments). Error bars display standard error of mean. *p* values were calculated using Student’s *t*-test (* *p* ≤ 0.05, ** *p* ≤ 0.01, *** *p* ≤ 0.001, and **** *p* ≤ 0.0001).

Following this validation of engineered CRISPR/Cas9-SAM cells for singleplex FX overexpression, we sought to assess the capabilities of these cells for simultaneous multiplex gene activation. The FBN peptide is comprised of three sub-units encoded by the *FBA*, *FBB*, and *FBG* genes, respectively. For this we designed, built, and delivered gRNAs targeting each gene and applied them to each of our transgenic cell lines. Subsequent qRT-PCR analysis showed supraphysiological levels of each gene compared with untreated controls (Figure 3A–D). 

Having observed overexpression of *FBA*, *FBB*, and *FBG* genes at the mRNA level, we then analyzed the cellular lysate and supernatant for trimeric FBN protein by ELISA (Figure 3E). For this, we employed an ELISA specifically designed to detect the formed trimeric complex. With this method, we observed yields of 401 +/− 64 ng/mL in HEP-g2 cells, 41 +/− 13 ng/mL in HKB11 cells, 15 +/− 2 ng/mL in HEK293 cells, and 2 +/− 2 ng/mL in SK-HEP1 cells that were predominantly prevalent as a secreted product (Figure 3E). These data support our hypothesis that the supraphysiological multiplexing gene expression mediated by transgenic human cells stably expressing the CRISPR/Cas9-SAM system translates into corresponding protein expression. 

### 2.3. Simplified Plasmid Engineering for CRISPR/Cas9-SAM Cell Line Transcriptome Modulation

**Figure 4:**
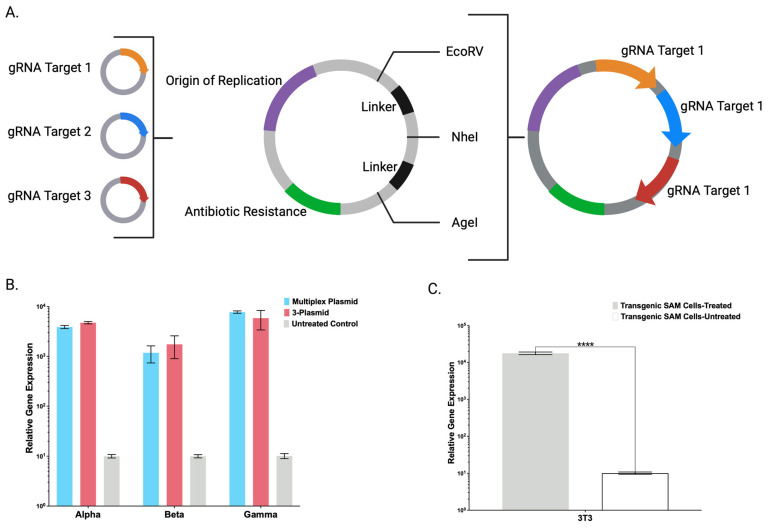
CRISPR/Cas9-SAM Adaptability and Tools for Expanded Applications. (**A**) Illustration displaying the design of a universal entry plasmid capable of delivering up to three individual gRNAs with adaptability to a unique target(s) via insertion into three unique single-cutter restriction endonuclease sites. (**B**) mRNA-based gene expression analysis of *FBA*, *FBB*, and *FBG* by qRT-PCR in transgenic HKB11 cells stably expressing dCas9-VP64 and λ-p65-HSF1 SAM system components treated with the targeting gRNAs delivered via a single multiplexing plasmid, three pooled individual plasmids, or left untreated. (**C**) mRNA-based gene expression analysis of murine *coagulation factor X* by qRT-PCR in transgenic NIH/3T3 cells stably expressing dCas9-VP64 and λ-p65-HSF1 following treatment with two gRNAs targeting murine *factor X*. (n = 3 independent experiments). Error bars display standard error of mean. *p* values were calculated using Student’s unpaired (**** *p* ≤ 0.0001).

Having shown supranormal gene and protein expression in each of the four transgenic human cell lines, we next took steps to streamline the CRISPR/Cas9-SAM components as a modular platform for gRNA cloning and expression. We developed a universal entry plasmid containing unique restriction enzyme sites separated by linkers enabling rapid cloning and expression of multiple gRNAs (Figure 4A). To validate this plasmid, we tested three gRNAs encoded on separate plasmids compared with an “all-in-one” plasmid expressing the same gRNAs targeting the *FBA*, *FBB*, and *FBG* genes (Figure 4B). We applied these plasmids in our transgenic HKB11 cells, and qRT-PCR analysis revealed a >100-fold increase in each FBN gene compared with the untreated control. Expression levels were similar between the three-plasmid and the all-in-one approaches (Figure 4B). This universal entry plasmid enables the delivery of multiple gRNAs for multiplexing gene targeting, making it a useful tool for facile SAM delivery in cells.

### 2.4. Trans-Species CRISPR/Cas9-SAM Application

To further extend the impact of the integrated CRISPR/Cas9-SAM system, we sought to establish proof of principle for trans-species application. Our rationale was that in addition to biotherapeutic human peptides, a multitude of preclinical and biological areas of investigation employ animal models and species-specific recombinant peptides aid in these efforts [21]. Therefore, we used viral transgenesis (Figure 1B) and integrated the CRISPR/Cas9-SAM system into the genome of the NIH/3T3 mouse embryonic fibroblast cell line. We then designed, built, and delivered gRNAs targeting murine *coagulation factor X* to these transgenic NIH/3T3 cells. Analyzing *FX* transcripts via qRT-PCR, we observed a 1752-fold increase in expression compared with the untreated control. These data extend the functionality of the CRISPR/Cas9-SAM system for targeted gene overexpression to multiple mammalian cell lines (Figure 4C). 

## 3. Discussion

Nucleic acids can be delivered to cells in vitro and serve as a template for recombinant gene expression and peptide production. The method and efficiency of nucleic acid template delivery, the constituents of the gene and protein, and the capability of a candidate cell type to facilitate efficient production, particularly for mediating post-translational modifications, are key considerations for designing and implementing recombinant strategies.

Exogenous nucleic acids, in the form of DNA or RNA, can be introduced into cells to direct recombinant expression. RNA is immediately available for transcription leading to high-level expression and contains no extraneous nucleic acid components (e.g., plasmid backbone). However, RNA is progressively degraded making it poorly suited for engineering cells for continuous protein production. DNA templates are often carried on plasmids; small circular DNA molecules containing full-length cDNAs and gene regulatory elements. Plasmids are central to recombinant engineering and upon delivery in vitro can exist either as an extrachromosomal episome or as an integrated molecule into the genome. Like RNA, episomal DNA is diluted as the cell divides and represents a transient protein production strategy. Integrated DNA offers the ability to continuously express the candidate gene(s) as part of a stable recombinant production platform. Recombinant nucleic acid cargo can include single or multi-gene cDNAs for producing monomeric or multimeric peptides, respectively. These architectural considerations directly impact the type and amount of nucleic acid that must be delivered to cells. 

The structure and function of candidate recombinant peptides are impacted by post-translational modifications. PTMs include glycosylation, acetylation, phosphorylation, and disulfide bond formation and the type, number, and location of these modifications are largely unique to a given species [22]. Historically, *Escherichia coli, Saccharomyces cerevisiae*, and Chinese hamster ovary (CHO) cells have served as leading options for recombinant strategies [23]. *E. coli*, a Gram-negative prokaryotic bacterium, is characterized by low-cost culture, rapid growth tolerant of high-density conditions, and simple gene transfer leading to high-yield protein production. Despite these benefits, *E. coli* show a diminished ability to mediate PTMs found in human peptides including glycosylation, sulfation, and the formation of disulfide bonds [24,25]. These limitations can impair secretion and lead to the formation of inclusion bodies, protein aggregates typically formed in the cytoplasm during the overexpression of proteins, which necessitate additional downstream processing including cell lysis steps to release retained peptides, purification to remove potentially harmful cellular debris, and labor and fiscally intensive approaches to purify and refold peptides [8,24]. These considerations make eukaryotic organisms desirable. 

Yeast such as *Saccharomyces cerevisiae* exhibit many of the advantages of bacterial platforms such as rapid growth, tolerance of high-density conditions, and efficient gene transfer. Compared with prokaryotes, yeast possess an enhanced capacity for depositing PTMs that mimic but do not fully recapitulate all higher level eukaryotic (e.g., human) PTMs [25]. As a primary example, glycosylation carried out in S. *cerevisiae* results in glycans significantly different from those found in most mammals due to the hyper-addition of mannose residues [26]. These residues can cause retention and partial degradation of peptides leading to suboptimal purity and contamination of end products with difficult-to-remove degradation products [26].

CHO cells have been widely employed for peptide production as a mammalian cell type. Like bacteria and yeast, they can be propagated at high density for high-yield protein production. Stable CHO engineering practices rely on the initial development of an expression vector that integrates into the genome [17]. Plasmid DNA integration occurs at a low frequency and requires labor-intensive clonal selection to identify transgenic clones [17,27]. A key benefit of CHO cells is their ability to mediate PTMs that are biosimilar to those found on human peptides. Yet these PTM include the addition of N-glycolylneuraminic acid (Neu5Gc), a form of glycosylation not found in humans [11,12]. This is a critical consideration given that more than 50% of human proteins are glycosylated, most people have anti-Neu5Gc antibodies, and many therapeutic peptides are often given in multiple doses potentially contributing to immune sensitization [11,28]. The inability of prokaryotic and non-human eukaryotic cellular platforms to uniformly mediate the production of fully humanized proteins makes human cell systems highly desirable for recombinant therapeutic protein production. 

We sought to define and optimize the conditions for human cell production of monomeric and multimeric proteins using coagulation factor X (FX) and fibrinogen (FBN) as proof of concept. Various hematological dyscrasias and coagulopathies often result in an increased propensity for clinically significant bleeding episodes that require the administration of hemostatic agents. FBN and FX are among these and are frequently administered in bleeding episodes to achieve hemostasis [1,2,3,4,5]. We pursued a strategy for the production of these proteins using a CRISPR gene activation approach where strong transcriptional activator elements were embedded in the genome of human cell lines: HEK293, HKB11, SK-HEP1, and HEP-g2 that have all demonstrated potential for recombinant peptide production including coagulation factors [13,14,15,16]. HEK293 cells are an immortalized embryonic kidney cell line known for their ease of culture and efficient transfection [29]. HKB11 cells are a hybrid of HEK293 cells and human Burkitt lymphoma B cells that, with an increased capacity for protein expression, typically exceed yields of HEK293 cells when utilized for coagulation factor production [14]. Immortalized hepatocyte SK-HEP1 and HEP-g2 cells have also shown promise for recombinant protein production and were used by Corrêa de Freitas and colleagues for coagulation factor production [15]. We reasoned that these cell types would benefit from a cross-platform comparison to define the optimal cell type and engineering approaches for recombinant peptide production using FX and FBN as candidates.

HEK293 cells have been employed by research groups lead by Ebert and Popovic for the production of FX and FBN, respectively [13,30]. Ebert pursued a transient plasmid-based approach for monomeric FX production [30]. Likewise, Popovic also employed plasmids carrying the individual *alpha (FBA)*, *beta (FBB)*, *and gamma (FBG)* chains of FBN or an all-in-one single plasmid [13]. Generation of a single plasmid with all three cDNAs necessitated the assembly of each chain-specific cDNA with unique promoters to avoid repetitive sequences that could lead to undesirable recombination [13]. The resulting plasmid of ~17,000 nucleotides displayed increased cellular toxicity potential due to the delivery of large genetic constructs. This consideration and the transient expression profile of episomal plasmids has prompted alternative approaches including efforts to introduce transgenes into the genome. 

Preceding Popovic, Hirashima employed a single plasmid containing *FBA*, *FBB*, and *FBG* cDNAs that was linearized and delivered to CHO cells for stable genomic insertion [10]. Subsequent clonal isolation and evaluation allowed for expansion of a transgenic cell line capable of producing FBN. Because plasmids integrate at low frequencies and contain the surrounding bacterial backbone elements that can contribute to silencing, we hypothesized that lentiviral transgenesis could be employed to efficiently engineer transgenic human cell lines expressing the CRISPR/Cas9-synergistic activator mediator (SAM) system. SAMs comprised of dCas9-VP64 and λ-p65-HSF1 were assembled into two lentiviral plasmids and packaged in VSV-G envelope pseudotyped viral particles. Stable HEK293, HKB11, SK-HEP1, and HEP-g2 cell lines were rapidly obtained (approximately two weeks) (Figure 1B). To characterize and validate our cell lines and approach, we delivered gRNAs targeting *FX* or *FBN* to our cells and demonstrated gene expression upregulation by qRT-PCR (Figure 2A and Figure 3A–D). 

Toward further validating our transgenic cell lines that showed robust mRNA overexpression (Figure 2, Figure 3 and Figure 4), we employed ELISA and Western blotting. ELISA confirmed the production and secretion of both FX and FBN (Figure 2B and Figure 3E), which was observed as a majority fraction in the cellular supernatant. Toward determining the proper assembly of the peptide, we likewise employed ELISA and Western blotting. Our choice for FBN characterization was carefully considered as proper FBN assembly requires multimeric protein folding mediated by the formation of 29 disulfide bonds and our ELISA is specially designed to detect the full trimeric complex [13]. Accordingly, we observed supranormal FBN protein levels in the supernatant (Figure 3E). In contrast with the FBN ELISA, the FX ELISA does not discriminate FX peptides that are known to exist as pre-/pro-isoforms with significant PTMs. Therefore, we employed Western blotting and chose our HEK293 and HKB11, which do not possess significant innate FX production levels (Figure 2A). In controls and SAM cells, we observed equal amounts of total protein (Figure 2D). Upon interrogation with an FX antibody, we observed bands consistent with FX production as a pre/pro and fully processed peptide (Figure 2B). 

Toward streamlining the engineering and maximizing the adaptability of the CRISPR/Cas9-SAM system, we also created a broadly applicable universal entry plasmid into which candidate gRNA(s) can be rapidly inserted (Figure 4A). A single plasmid with multiple gRNAs was comparable to gRNAs delivered on individual plasmids (Figure 4B). Decreasing the amount of DNA will preserve maximal cell viability that can be diminished by large amounts of ectopic DNA [13]. Having demonstrated defined CRISPR/Cas9-SAM parameters in human cells, we generated a transgenic murine cell line that showed supraphysiological FX overexpression (Figure 4C) and expanded the application for human and murine cell engineering. 

**Figure 5:**
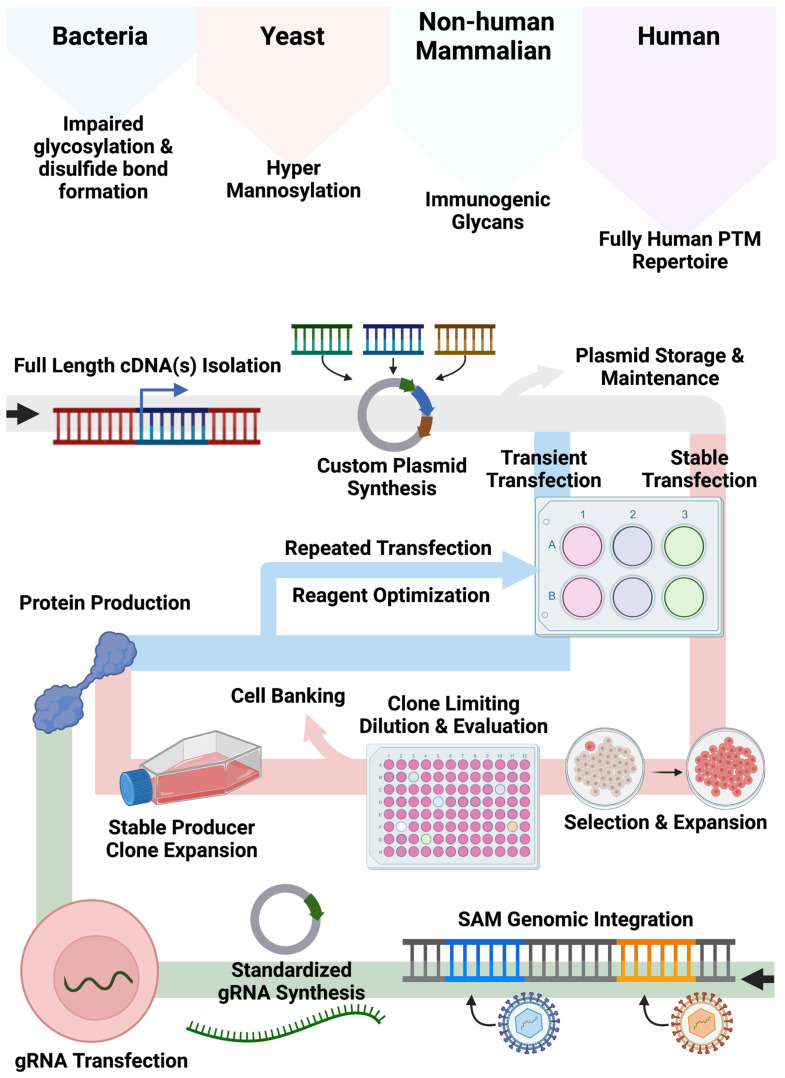
Plasmid-based and SAM-based Recombinant Protein Engineering Approaches. Illustration of the engineering methods to produce recombinant proteins using plasmid-based and SAM-based engineering approaches.

Our bespoke cell lines augment and expand current approaches for recombinant protein production employing non-human cell systems and/or plasmid-based gene expression (Figure 5). Bacteria, yeast, and non-human mammalian cells can show altered glycan and other PTMs compared with human cell types. While the application of human cells can mitigate aberrant PTM deposition patterns, associated expression approaches often rely on large plasmids encoding expression components including a promoter, candidate cDNA(s), and a polyadenylation signal required for recombinant engineering. As Popovic and Hirashima detailed, cDNAs for multimeric proteins can be generated and delivered singly or in tandem [10,13]. While effective, this customized approach requires quality assurance and control and gene transfer optimization to fully characterize the conditions for plasmid delivery for transient or stable production. Accordingly, we sought to create human cell lines that could be induced to upregulate genes from their native locus regardless of baseline expression by embedding strong transcriptional elements in their genome. In our design, gene transcription is triggered upon delivery of a standardized small (<4 kb) gRNA plasmid that can be rapidly produced and characterized. This design feature bypasses the need for customized full-gene cloning/synthesis and assembly of large plasmids with designer regulatory elements. 

In summation, our engineering approach for CRISPR/Cas9-SAM system integration into human and murine cells generates cell lines enabling trans-species singleplex and multiplex transcription and translation modulation. HEK293, HKB11, and SK-HEP1 that do not normally express significant levels of FBN can be induced to do so via SAM upregulation. Alternatively, FBN expressed at high basal levels in HEP-g2 can be further potentiated with SAMs representing an approach to maximize the innate properties of a cell type of interest to produce protein with SAMs. Cells such as these hold potential for biotherapeutic human peptide production and transcriptional modeling. 

## 4. Materials and Methods

### 4.1. SAM Vector and Lentiviral Vector Assembly

Plasmids carrying dCas9-VP64, Lambda-p65-HSF1, and gene-specific gRNAs were assembled as previously described by Gibson Assembly using a HiFi DNA Assembly Master Mix from New England BioLabs (Ipswich, MA, USA) per the manufacturer’s instructions. In a similar fashion, the SAM components dCas9-VP64 and Lambda-p65-HSF1 were shuttled into lentiviral vectors harboring puromycin or hygromycin B antibiotic resistance genes respectively driven by the MND promoter. Antibiotic-resistant genes were linked to SAM components via a self-cleaving T2A sequence. Oligonucleotides for cloning were synthesized by IDT (Coralville, IA, USA).

### 4.2. Cell Culture and Transfection Reagents

HEK293 cells and 3T3 cells, grown in DMEM complete media, were obtained from ATCC (CRL-1573 and CRL-1658) (Manassas, VA, USA). SK-HEP-1 cells and HEP-g2 cells, grown in EMEM complete media, were obtained from ATCC (HTB-52 and HB-8065). HKB11 cells, grown in DMEM/F12 complete media, were obtained from ATCC (CRL-12568). Complete media contained 5% fetal bovine serum from Gibco (Grand Island, NY, USA) and was supplemented with penicillin/streptomycin from Sigma-Aldrich (Burlington, MA, USA). The adherent cells were grown as a monolayer in T75 cm^2^ Corning (Corning, NY, USA) cell-culture-treated flasks and detached with Trypsin-EDTA solution (0.25%) obtained from Invitrogen (Waltham, MA, USA). Transient transfections utilized Lipofectamine 2000 (HEK293 and 3T3) or Lipofectamine 3000 (HKB11, SK-HEP1, and HEP-g2) from Invitrogen seeding 100,000 cells per well in a Corning 24-well plate. All cells were cultured at 5% CO2 and 37 °C. 

### 4.3. SAM Lentivirus Production and Transduction

A third-generation four-plasmid packaging system was utilized to produce lentivirus as previously described in HEK293 cells. Briefly, viral and cargo plasmids were transfected into cells using Lipofectamine 2000, and subsequent vesicular stomatitis virus glycoprotein G (VSV-G)-enveloped viral particles were harvested and concentrated using a Lenti-X Concentrator from Clontech (Mountain View, CA, USA). Candidate cell lines were transduced and expanded before selection with puromycin or hygromycin B from Sigma-Aldrich (Burlington, MA, USA).

### 4.4. Quantitative RT-PCR

Total mRNA was isolated using the RNeasy Plus kit from Qiagen (Hilden, Germany). Equal amounts of RNA were reverse-transcribed Superscript VILO-IV from Invitrogen (Waltham, MA, USA). Gene expression analysis was carried out on a QuantStudio 3 real-time PCR system from Applied Biosystems (Waltham, MA, USA). Taqman gene expression master mix and probes were also from Applied Biosystems. Expression data were normalized using GAPDH using the 2-ΔΔCT method. See Appendix A for a complete list of probes. 

### 4.5. Western Blotting

Equal amounts of conditioned media were resolved by 4–12% gradient SDS–polyacrylamide gel electrophoresis in a ThermoFisher Scientific (Waltham, MA, USA) mini gel tank run at a constant 120 volts. The precision plus protein standard from BioRad (Hercules, CA, USA) was used to track protein size. After electrophoresis, the proteins were transferred to a nitrocellulose membrane using the Invitrogen (Waltham, MA, USA) iBlot 2 gel transfer system. Membranes were stained with Ponceau S from ThermoFisher Scientific (Waltham, MA, USA) according to the manufacturer’s instructions to gauge protein transfer efficiency and confirm equal protein loading before the stain was removed. Membranes were then blocked for 1 h with phosphate-buffered saline–Tween 20 (PBS-T) containing 10% non-fat dry milk and incubated overnight with the primary antibody (ab79929) from Abcam (Waltham, MA, USA) in PBS-T with 10% non-fat dry milk. After washing, the membranes were incubated with a specific HRP-conjugated secondary antibody (sc-2357) from Santa Cruz Biotechnology (Dallas, TX, USA) in PBS-T containing 10% non-fat dry milk and antibody binding was detected by SuperSignal West Pico Plus chemiluminescent substrate from ThermoFisher Scientific (Waltham, MA, USA) according to the manufacturers’ instructions. All images were taken with the BioRad ChemiDoc MP Imaging System version 3.0.1.

### 4.6. Protein Quantification

Conditioned media and cell lysate were collected from cells. Cells were lysed in Reporter Lysis Buffer from Promega (Madison, WI, USA) for 30 min before shearing through a 27.5-gauge needle. Both cell lysate and media were cleared of cellular debris by centrifugation before analysis. The ELISA kit for FX (ab108832) and FBN (ab241383) was purchased from Abcam (Cambridge, UK). Analysis was conducted on a Tecan Infinite Microplate reader (Männedorf, Switzerland) using Magellan software version 7.5. 

### 4.7. Graphing, Statistical Analysis, and Images

All graphs were constructed using GraphPad Prism software v.8. Statistical analyses were performed using unpaired two-sided t-tests to compare the treated and untreated cells in transfection experiments. Images were constructed and formatted using BioRender 2023. 

## Data Availability

Additional data files are available upon request.

## Appendix A

**Table A1:** Complete list of qRT-PCR probes used for detection of transcripts of interest.

Gene	qRT-PCR Probe
Fibrinogen alpha	Hs00241027_m1
Fibrinogen beta	Hs00170586_m1
Fibrinogen gamma	Hs00241037_m1
Factor X (human)	Hs00984442_m1
Factor X (mouse)	Mm00484177_m1
GAPDH	Hs02758991_g1
